# Cancer cell-derived exosomal circUSP7 induces CD8^+^ T cell dysfunction and anti-PD1 resistance by regulating the miR-934/SHP2 axis in NSCLC

**DOI:** 10.1186/s12943-021-01448-x

**Published:** 2021-11-09

**Authors:** Shi-Wei Chen, Shu-Qiang Zhu, Xu Pei, Bai-Quan Qiu, Dian Xiong, Xiang Long, Kun Lin, Feng Lu, Jian-Jun Xu, Yong-Bing Wu

**Affiliations:** Department of Cardiothoracic Surgery, the Second Affiliated Hospital of Nanchang University, 1 Ming de Road, Nanchang, 330000 People’s Republic of China

**Keywords:** circUSP7, NSCLC, Exosome, Anti-PD1, miR-934, SHP2

## Abstract

**Background:**

CD8^+^ T cells play a critical role in the innate antitumour immune response. Recently, CD8^+^ T cell dysfunction has been verified in various malignant cancers, including non-small cell lung cancer (NSCLC). However, the molecular biological mechanisms of CD8^+^ T cell dysfunction in human NSCLC are still unclear.

**Methods:**

The expression of circular ubiquitin-specific protease-7 (circUSP7) in NSCLC tissues, exosomes, and cell lines was detected by quantitative real-time polymerase chain reaction (qRT-PCR). Exosomes were isolated from the culture medium of NSCLC cells and the plasma of NSCLC patients using an ultracentrifugation method and the ExoQuick Exosome Precipitation Solution kit. The exosomes were then characterized by transmission electronic microscopy (TEM), NanoSight and western blotting. The role of circUSP7 in CD8^+^ T cell dysfunction was assessed by enzyme-linked immunosorbent assay (ELISA). In vivo circular RNA (circRNA) precipitation (circRIP), RNA immunoprecipitation (RIP), and luciferase reporter assays were performed to explore the molecular mechanisms of circUSP7 in CD8^+^ T cells. In a retrospective study, the clinical characteristics and prognostic significance of circUSP7 in NSCLC tissues were determined.

**Results:**

The expression levels of circUSP7 were higher in human NSCLC tissues than in matched adjacent nontumour tissues. Increased levels of circUSP7 indicate poor clinical prognosis and CD8^+^ T cell dysfunction in patients with NSCLC. The circUSP7 found in NSCLC patient plasma is predominantly secreted by NSCLC cells in an exosomal manner, and circUSP7 inhibits IFN-γ, TNF-α, Granzyme-B and Perforin secretion by CD8^+^ T cells. Furthermore, circUSP7 inhibits CD8^+^ T cell function by upregulating the expression of Src homology region 2 (SH2)-containing protein tyrosine phosphatase 2 (SHP2) via sponging miR-934. Finally, we show that circUSP7 may promote resistance to anti-PD1 immunotherapy in NSCLC patients.

**Conclusions:**

Exosomal circUSP7 is predominantly secreted by NSCLC cells and contributes to immunosuppression by promoting CD8^+^ T cell dysfunction in NSCLC. CircUSP7 induces resistance to anti-PD1 immunotherapy, providing a potential therapeutic strategy for NSCLC patients.

**Supplementary Information:**

The online version contains supplementary material available at 10.1186/s12943-021-01448-x.

## Introduction

Lung cancer is a major malignant tumour and the leading cause of cancer-related deaths worldwide [1]. The prognosis of lung cancer is influenced by important factors that result in treatment failure, including cancer invasion and metastasis. Non-small cell lung cancer (NSCLC) is the leading cause of cancer-related deaths and the major pathological type of lung cancer in China. Recently, the use of programmed death receptor-1 (PD1) antibodies has decreased tumour progression and provided long-term clinical benefits in patients with NSCLC. Unfortunately, the majority of patients inevitably acquire resistance after several cycles of treatment [2, 3]; therefore, it is crucial to actively explore the mechanisms of anti-PD1 resistance in NSCLC.

Src homology region 2 (SH2)-containing protein tyrosine phosphatase 2 (SHP2), which is encoded by the PTPN11 gene, is universally expressed in various mammalian cells. SHP2 plays diverse roles in many cellular processes. A number of studies have demonstrated an oncogenic role of SHP2 in diverse types of cancer, and the upregulated expression of SHP2 in NSCLC is associated with invasive behaviour and poor prognosis via multiple mechanisms; for example, the growth of KRAS-mutant NSCLC is inhibited by inhibition of SHP2 in vivo [4]. When PD1 binds to its physiological ligand (PD-L1 or PD-L2), it suppresses the activation and function of T cells through the recruitment of SHP2, which dephosphorylates and inactivates Zap70, a major integrator of TCR-mediated signalling [5–9]. These results suggest that SHP2 may be a novel target of antineoplastic drugs. Indeed, an allosteric inhibitor of SHP2 has been confirmed to inhibit cancer cell growth in vivo and in vitro [10]. SHP2 is usually overexpressed in NSCLC; however, its association with the progression and development of the disease as well as the molecular mechanism by which SHP2 contributes to tumour cell growth remain largely unclear.

Circular RNAs (circRNAs), endogenous noncoding RNAs derived from a single exon or multiple exons, are present in the cytoplasm of cells of a variety of organisms. Tumorigenesis and cancer progression are generally affected by circRNAs that have conserved, covalently immobilized closed-loop structures and are more difficult to degrade than linear RNAs with 5′ caps and 3′ poly(A) tails [11–13]. For example, we previously reported that circMET promotes NSCLC cell proliferation, metastasis, and immune evasion by regulating the miR-145-5p/CXCL3 axis [14]. Furthermore, Zhang et al. demonstrated that cancer cell-derived exosomal circular ubiquitin-like with PHD and ring finger domain 1 RNA (circUHRF1) induces natural killer cell exhaustion and may cause resistance to anti-PD1 therapy in hepatocellular carcinoma patients [15]. A few circRNAs have been proven to regulate carcinoma progression; however, the function of circRNAs in NSCLC remains far from clear.

Exosomes are membrane-bound extracellular vesicles (EVs) that are produced in the endosomal compartment of most eukaryotic cells [16–18]. Intercellular communication via exosomes is a critical mediator of biological processes [19–21]. In recent years, an increasing number of studies have reported that exosomes perform important biological functions in the body, especially in the occurrence and development of tumours and the presence of circRNAs [22, 23]. Long noncoding RNAs (lncRNAs) and mircoRNAs (miRNAs) in exosomes are closely related to the immunosuppression, migration, proliferation, and invasion of cancers [24–27]. However, the potential roles and expression patterns of circRNAs in both tumours and plasma exosomes of NSCLC patients have not been definitively elucidated.

## Materials and methods

### Cell culture and transfection

The NSCLC cell lines NCI-H460, NCI-H1299, A549, PC9, and 95D and the normal human bronchial epithelial cell line HBE were purchased from the Cell Bank of the Chinese Academy of Sciences (Shanghai, China). RPMI-1640 or DMEM (HyClone, Logan City, USA) supplemented with 10% foetal bovine serum (FBS) (Gibco, USA) and 1% penicillin-streptomycin solution (Yeasen, China) was used to culture the NSCLC cells at 37 °C in a humidified atmosphere containing 5% CO_2_. All the lentiviral vectors used in this study were obtained from Genomeditech (Shanghai, China). The circUSP7-overexpression and circUSP7-short hairpin RNA (shRNA) lentiviral vectors were transfected into NSCLC cells according to the manufacturer’s instructions.

### Clinical specimens

A total of 126 pairs of NSCLC tissue samples and corresponding adjacent nontumour tissue samples, as well as preoperative and postoperative plasma samples, were obtained from patients who underwent surgery at the Second Affiliated Hospital of Nanchang University. In addition, fresh normal plasma samples were collected from 10 healthy age- and sex-matched volunteers at the Second Affiliated Hospital of Nanchang University. The detailed clinicopathological characteristics of the subjects are summarized in Table 1. This study was approved by the Ethical Review Committee of the Second Affiliated Hospital of NanChang University, and written consent was obtained from all the patients involved.

**Table 1:** Correlation between circ USP7 and clinical characteristics in 126 NSCLC patients

Variables	CircUSP7 expression level		***p*** value
	Low	High	
Age			
60<	31	34	0.722
60≥	32	29	
Gender			
Male	35	39	0.587
Female	28	24	
Smoking status			
Smokers	40	43	0.707
Nonsmokers	23	20	
Histological type			
Squamous cell carcinoma	18	21	0.700
Adenocarcinomas	45	42	
Tumor stage			
I-II	27	34	0.285
III-IV	36	29	
Tumor size			
≤ 3	36	21	0.012
> 3	27	42	
Lymph node metastasis			
Yes	20	34	0.019
No	43	29	
Intravascular cancer embolus			
Yes	17	29	0.041
No	46	34	
Differentiation			
Well and moderate	37	35	0.857
Poor	26	28	

### Exosome isolation and incubation with CD8^+^ T cells

Exosomes were isolated from the plasma of NSCLC patients and the culture medium of NSCLC cell lines by ExoQuick Exosome Precipitation Solution (SBI System Biosciences, Cat: EXOQ5A-1) according to the manufacturer’s instructions. Next, transmission electron microscopy (TEM) was used to examine the exosomes, as previously described. Exosomes (500 μg) derived from the NSCLC cell lines were placed into 12-well plates, and preactivated CD8^+^ T cells were independently added. After incubation for 24 h, flow cytometry and quantitative real-time polymerase chain reaction (qRT-PCR) were used to analyse CD8^+^ T cells.

### RNA isolation, qRT-PCR and western blot

Total RNA was isolated from tissue samples and cultured cells using TRIzol Reagent (Invitrogen, USA) according to the manufacturer’s instructions, and the samples were reverse transcribed into cDNA with a PrimeScript RT Reagent Kit (TaKaRa, Japan). qRT-PCR was performed with SYBR Green Real-time PCR Master Mix (Yeasen, Shanghai, China). Similarly, quantification of the levels of circRNAs and mRNAs was performed by normalization to the levels of glyceraldehyde-3-phosphate dehydrogenase (GAPDH), which was used as the internal reference gene. The relative RNA expression levels were analysed by utilizing the 2-ΔΔCt method. All primers were included in Additional File 1: Supplementary Table 1. For western blot analysis, the cells were lysed in radioimmunoprecipitation assay (RIPA) buffer (Yeasen, Shanghai, China), and the cell lysates were centrifuged for 15 min at 12,000 g (4 °C). The supernatants containing proteins were collected. Consequently, the collected proteins were added to a 10% SDS-PAGE gel and transferred to polyvinylidene fluoride (PVDF) membranes (Millipore) by using a semidry blotter. After blocking with 5% nonfat milk for 1 h at room temperature with shaking, the membranes were incubated overnight with the appropriate primary antibodies at 4 °C. After washing with TBST, horseradish peroxidase (HRP)-conjugated secondary antibodies (Cell Signaling Technology) were incubated with the membranes for 1 h at room temperature with shaking. The protein amounts on the membranes were visualized by densitometry using an enhanced chemiluminescence reagent (Amersham Pharmacia Biotech, Piscataway, NJ). An anti-GAPDH antibody (1:1000, Affinity, USA) served as the loading control. Each experiment was separately carried out in triplicate.

### Immunohistochemistry (IHC)

Tissue microarrays (TMAs) of 126 pairs of NSCLC tissues and adjacent peritumoural tissues were generated by Shanghai Biochip Co., Ltd. (Shanghai, China). The IHC assay was performed according to the protocol. Briefly, xylene and different concentrations of alcohol were used to soak all the paraffin NSCLC tissue TMAs for dewaxing and hydration; consequently, the TMAs were incubated in 3% H_2_O_2_ at room temperature for 30 min to block endogenous peroxidase activity. Then, 5% bovine serum albumin (BSA) (Yesen, Shanghai, China) was added and incubated for 1 h at room temperature to block the nonspecific binding sites. Subsequently, the primary polyclonal antibodies were added and incubated with the TMAs overnight at 4 °C, followed by incubation with the corresponding biotinylated secondary antibodies for 1 h at room temperature. The TMAs were washed with PBS after every incubation. Next, the chromogen reaction was detected by staining the slides with diaminobenzidine (DAB)-H_2_O_2_ (GeneTech, Shanghai, China) and lightly counterstaining with haematoxylin, following the manufacturer’s protocol. Finally, the slides were covered with a cover slip, sealed with neutral balsam (Yeasen, Shanghai, China) and visualized under a fluorescence microscope.

### CD8^+^ T cell isolation

Human CD8^+^ T cells were isolated and purified from healthy donor peripheral blood mononuclear cells (PBMCs) by an Easy-Sep™ Direct Human CD8^+^ T Cell Isolation Kit (STEMCELL Technologies). For CD8^+^ T cell activation and proliferation, human CD8^+^ T cells were seeded into 24-well plates, and anti-CD3/anti-CD28 antibodies (BD Biosciences) were added and incubated for 48 h. The miRNA mimic or scramble RNA was transfected into preactivated CD8^+^ T cells using a human T cell transfection kit (Lonza).

### CircRNA in vivo precipitation (circRIP), RNA immunoprecipitation (RIP), and luciferase reporter assays

In vivo circRIP, RIP, and luciferase reporter assays were performed as described [14]. For the circRIP and RIP experiments, biotin-labelled circUSP7 and negative control (NC) probes were synthesized by GenePharma (Shanghai, China). The circRIP assay was performed as previously described [14], and the RIP assays were performed using a Magna RIP RNA Binding Protein Immunoprecipitation Kit (Millipore, Cat: 17–770) according to the manufacturer’s instructions. Additionally, luciferase activity was determined using a dual-luciferase reporter, and the wild-type SHP2 3′ untranslated region (UTR) and circUSP7 sequences were cloned into a pLG3 plasmid. The mutant SHP2 3′ UTR and circUSP7 pLG3 plasmids were generated using a mutagenesis kit (Qiagen, USA, Cat: 200521) according to the manufacturer’s instructions. Healthy donor-derived CD8^+^ T cells were seeded into 96-well plates and cotransfected with a luciferase reporter vector and the miR-934 mimics or NC using Lipofectamine 2000 transfection reagent (Thermo Fisher, Cat: 11668–019). After 48 h, the firefly and Renilla luciferase activities were quantified with the Dual-Luciferase Reporter Assay System (Promega, USA, Cat: E1910).

### RNA pulldown assay

The pulldown assay was performed as previously described [14]. Briefly, to generate probe-coated beads, the biotinylated circUSP7 probe, biotinylated circANRIL probe, and biotinylated NC probe (GenePharma, China) were incubated with M-280 streptavidin magnetic beads (Invitrogen, USA, Cat: 11205D) at room temperature for 2 h. Then, approximately 1 × 10^7^ CD8^+^ T cells were harvested, lysed, and sonicated, and these lysates were incubated with the probe-coated beads at 4 °C overnight. Subsequently, the RNA complexes bound to the beads were eluted and extracted with an RNeasy Mini Kit (QIAGEN, USA, Cat: 74104) and analysed by qRT-PCR.

### Enzyme-linked Immunosorbent Assay (ELISA)

The concentrations of IFN-γ, TNF-α, Granzyme-B and Perforin produced by CD8^+^ T cells were measured by IFN-γ, TNF-α, Granzyme-B and Perforin ELISA kits (eBioscience, USA, Cat no.: KHC4021, Cat no.: BMS223HS, Cat no.: BMS2027 and Cat no.: BMS2306) in accordance with the manufacturer’s guidelines.

### Patient-derived Xenografts (PDX)

The protocols carried out to generate the PDX mice were approved by the Animal Experimentation Ethics Committee of Zhongshan Hospital, Fudan University (permit ID number: Y2016–025) and were previously established and described [28]. Written informed consent was provided by the patients or their legal guardians (the Second Affiliated Hospital of Nanchang University and Zhongshan Hospital of Fudan University). For transplantation, fresh tumour tissues from the PDX mice were chopped with surgical scissors, and the chopped tumour tissues were resuspended in 100–200 μl of a 1:1 v/v mixture of cold DMEM (HyClone, Logan City, USA) and kept on ice until transplantation. For the transplantation of intact cell fragments, a small incision was made in the skin of the flank, and then, the fragments were inserted using forceps. To maintain NSCLC-PDX tumours in NSG mice, NSCLC tissues obtained from first-generation mice were serially transplanted into the next cohorts of mice.

### Humanized mouse generation

NSG mice were purchased from the Jackson Laboratory and bred and raised under specific pathogen-free conditions. New-born NSG mice (1 to 3 days old) were irradiated with 1 Gy and injected with 1–2 × 10^5^ CD34^+^ human haematopoietic progenitor cells. CD34^+^ cells were isolated from human foetal liver tissue, as previously described [29]. Then, the reconstitution of human immune system components in the peripheral blood of the humanized NSG (HuNSG) mice in each cohort was analysed 12 weeks after engraftment and prior to the beginning of the experiments.

### In vivo tumour growth and anti-PD1 experiments

The xenograft experiments in the HuNSG mice were approved by the Animal Experimentation Ethics Committee of Zhongshan Hospital, Fudan University (permit ID number: Y2016–025). We resuspended 2 × 10^5^ cells (per mouse) in 100 μl of PBS and injected them into the right flank to generate subcutaneous tumours. The tumour size was measured every 3 days. The mice were randomly assigned to four groups. Then, the mice received tail vein injections of Opdivo or its isotype control (100 μg per dose) three times a week for 2 weeks. The day that the mice received the first dose was considered day 1. At 30 days after the initial injection, we detected the expression levels of exosomal circUSP7 in the plasma derived from HuNSG mice after collecting blood from the tail vein. The tumour volume was calculated as (length x width^2^)/2. The animals were euthanized when the tumours reached a maximum of 2000 mm^3^ (*n* = 6). The tumour specimens were surgically removed, fixed, embedded in paraffin, and sectioned. The sections were used for haematoxylin and eosin (H&E) and IHC staining.

### Statistical analysis

The SPSS 20.0 software program (IBM SPSS, Chicago, IL, USA) was used for statistical analyses. Comparisons between two groups were conducted using Student’s t-tests. Correlation analyses between two continuous variables were determined using the Pearson correlation coefficient. Survival curves were estimated by the Kaplan-Meier method and evaluated by the log-rank test. *P* < 0.05 was considered statistically significant.

## Results

### Characteristics of USP7-derived circRNAs released via exosomes in NSCLC

The relationship between USP7 and cancer cell invasion is known. Increasing numbers of studies have reported that high USP7 expression participates in the progression of multiple cancers, including NSCLC [30–33].

**Fig. 1:**
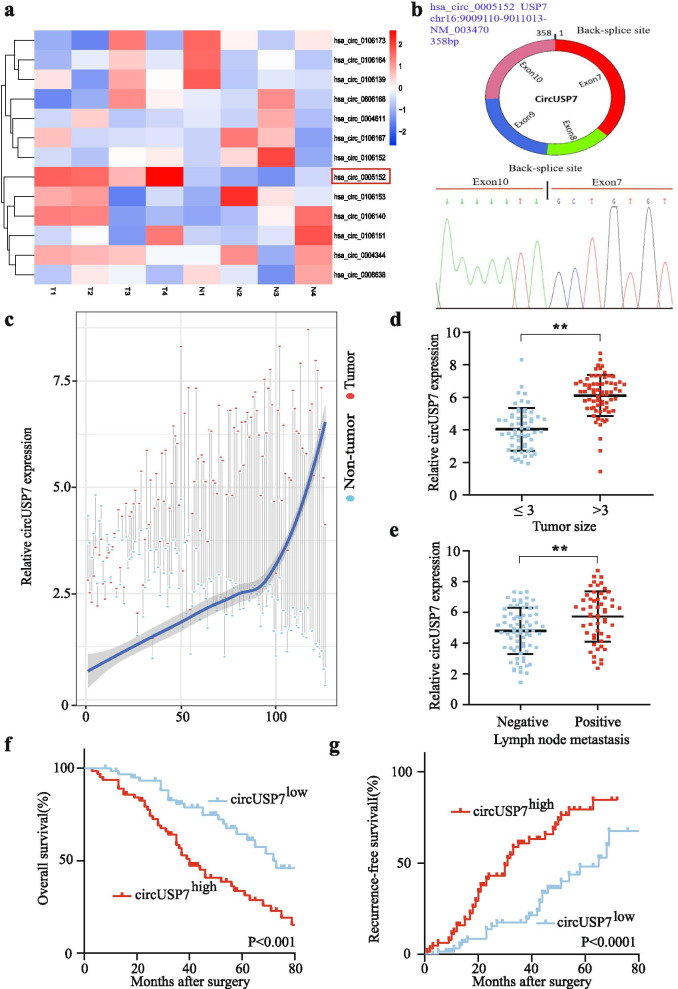
CircUSP7 is upregulated in NSCLC tissues. **a** Heatmap showing USP7 gene-derived circRNAs in NSCLC tissues compared with those in matched adjacent nontumour tissues, as analysed by qRT-PCR. **b** Diagram of the structure of circUSP7. **c** Differential expression of circUSP7 in the NSCLC tissues and adjacent nontumour tissues of 126 patients. **d** A total of 126 patients were divided into the ≤3 cm and >  3 cm size groups. The circUSP7 expression in each group is shown in the diagram. **e** A total of 126 NSCLC patients were divided into groups negative for lymph node metastasis or positive for lymph node metastasis. The diagram shows circUSP7 expression in each group. **f** and **g** Kaplan-Meier analysis of the OS and recurrence of 126 patients with NSCLC according to circUSP7 expression (log-rank test). The data are presented as the mean ± SD of three independent experiments. **P < 0.01, ***P < 0.001

Here, 13 USP7 gene-derived circRNAs were analysed by inspecting the circRNA sequencing data from StarBase v 3.0. The results showed that the expression levels of circUSP7 (hsa_circ_0005152) were significantly increased compared to those of other USP7-derived circRNAs in four paired NSCLC tissues. Importantly, the levels of hsa_circ_0005152 were downregulated in four of the adjacent nontumour lung tissues compared with those in the matched NSCLC tumour tissues (Fig. 1a). CircUSP7 consists of 4 exons and 358 nucleotides (Fig. 1b). Next, we examined the expression of circUSP7 in the tumour tissues and adjacent normal tissues of 126 NSCLC patients. The qRT-PCR analysis results demonstrated that the expression levels of circUSP7 in the NSCLC tissues were significantly increased compared to those in the adjacent normal tissues (Fig. 1c).

Furthermore, we explored the relationship between circUSP7 levels and the clinicopathological characteristics of 126 NSCLC patients, and the results are shown in (Tables 1 and Additional File 2: Supplementary Fig. 1). The results showed that NSCLC patients with higher circUSP7 expression had larger tumour sizes (Fig. 1d) and more positive lymph node metastases (Fig. 1e) than those with lower circUSP7 expression.

Moreover, we found that NSCLC patients with higher circUSP7 expression had a worse prognosis than those with lower circUSP7 expression (Fig. 1f and g). Multivariate analysis indicated that circUSP7 expression is an independent predictor of postoperative recurrence and overall survival (OS) (Tables 2 and 3). Taken together, the results described above suggested that circUSP7 expression is likely involved in the progression of NSCLC.

**Table 2:** Univariate and Multivariate Analyses of Factors Associated with Overall Survival

Factors	OS			
	Multivariate			
	Univariate ***p***	HR	95%CI	***P*** value
Sex (Female vs. Male)	0.645			NA
Age (years) (≤60 vs. > 60)	0.447			NA
Smoking status (Smokers vs. Nonsmokers)	0.896			NA
Histological type (SCC vs. Adenocarcinomas)	0.580			NA
Lymph node metastasis (Yes vs. No)	0.001	0.405	0.232–0.706	0.024
Differentiation (Well and moderate vs. Poor)	0.053			NS
Tumor size (diameter, cm) (> 3 vs. ≤3)	0.038			NS
TNM (III-IV vs. I-II)	0.276			NA
CircUSP7 expression (High vs. Low)	0.001	3.090	1.552–6.151	0.037

*OS* Overall survival, *NA* Not adopted, *NS* Not significantly, *SCC* Squamous cell carcinoma, *95%CI* 95% confidence interval, *HR* Hazard ratio; Cox proportional hazards regression model

**Table 3:** Univariate and Multivariate Analyses of Factors Associated with Cumulative Recurrence

Factors	Cumulative Recurrence			
	Multivariate			
	Univariate ***p***	HR	95%CI	***P*** value
Sex (Female vs. Male)	0.617			NA
Age (years) (≤60 vs. > 60)	0.987			NA
Smoking status (Smokers vs. Nonsmokers)	0.669			NA
Histological type (SCC vs. Adenocarcinomas)	0.535			NA
Lymph node metastasis (Yes vs. No)	0.001	0.394	0.225–0.689	0.043
Differentiation (Well and moderate vs. Poor)	0.012			NS
Tumor size (diameter, cm) (> 3 vs. ≤3)	0.103			NA
TNM (III-IV vs. I-II)	0.098			NS
CircUSP7 expression (High vs. Low)	0.006	2.537	1.300–4.949	0.051

*NA* Not adopted, *NS* Not significantly, *SCC* Squamous cell carcinoma, *95%CI* 95% confidence interval, *HR* hazard ratio; Cox proportional hazards regression model

### The expression level of plasma exosomal circUSP7 is upregulated in NSCLC patients

**Fig. 2:**
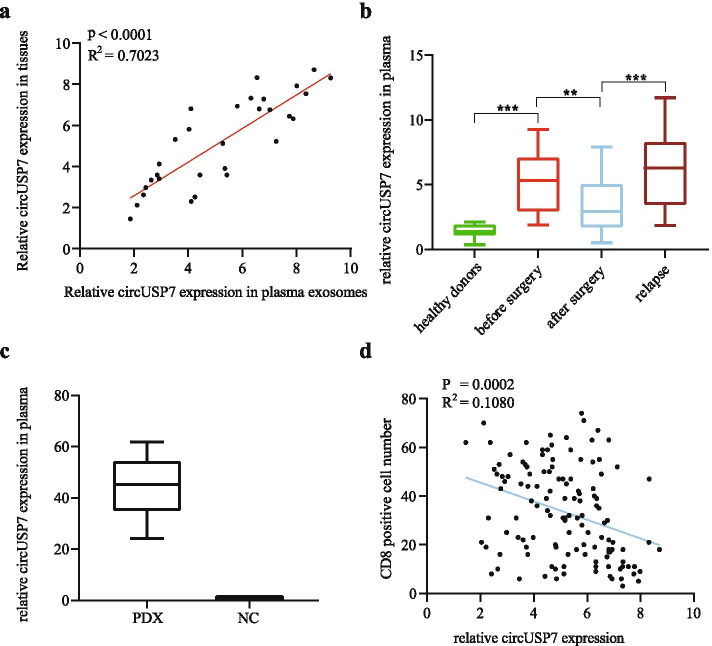
The expression level of plasma exosomal circUSP7 is upregulated in NSCLC patients. **a** The expression level of circUSP7 in the NSCLC tissues and preoperative plasma exosomes of NSCLC patients (R^2^ = 0.7023; *P* < 0.0001). **b** Exosomal circUSP7 expression in the plasma of healthy donors, patients before surgery, patients after surgery, and patients with recurrence. **c** The expression of plasma exosomal circUSP7 in the PDX or control group. **d** A negative correlation between the circUSP7 expression in plasma exosomes and the proportion of CD8^+^ T cells was observed in NSCLC patient tissues (R^2^ = 0.1080; *P* = 0.0002). The data are presented as the mean ± SD; *n* = 3, *P < 0.05, **P < 0.01

Above, we confirmed a negative correlation between circUSP7 expression and patient prognosis. Previous studies have proven that circRNAs can participate in cell-to-cell communication via exosomes. Thus, we detected the preoperative expression level of circUSP7 in the plasma exosomes of 30 patients who experienced postoperative recurrence and from whom cancer tissues were removed during surgery. The results of a scatter plot analysis also revealed a positive correlation between circUSP7 expression in the tumour tissues and circUSP7 expression in the plasma exosomes from patients with postoperative recurrence (Fig. 2a). Thus, we verified that circUSP7 is present in patient plasma exosomes. Then, plasma exosomes were extracted from 10 healthy donors and 30 patients with postoperative recurrence to detect the expression of circUSP7 by qRT-PCR. The plasma exosomal circUSP7 levels in the healthy controls were decreased compared with those in the NSCLC patients. Importantly, we found that the plasma exosomal circUSP7 levels were increased in NSCLC patients with recrudescence and decreased after the cancer tissues were removed during surgery (Fig. 2b). Mice with patient-derived xenografts (PDXs) were established using tissues from NSCLC patients, and the expression levels of plasma exosomal circUSP7 in the peripheral blood were detected. The results showed that the plasma exosomal circUSP7 levels in the PDX mice were increased compared with those in the control mice without tumours, indicating that plasma exosomal circUSP7 was commonly derived from NSCLC cells (Fig. 2c). Interestingly, in a scatter plot analysis, we found a negative correlation between circUSP7 expression in tumour tissues and the CD8^+^ T cell frequency in the tissues from 126 NSCLC patients (Fig. 2d). These results indicated that plasma exosomal circUSP7 may serve as a pivotal determinant of CD8^+^ T cell-related immune escape in NSCLC.

### CD8^+^ T cell function was inhibited by NSCLC cell-derived exosomal circUSP7

**Fig. 3:**
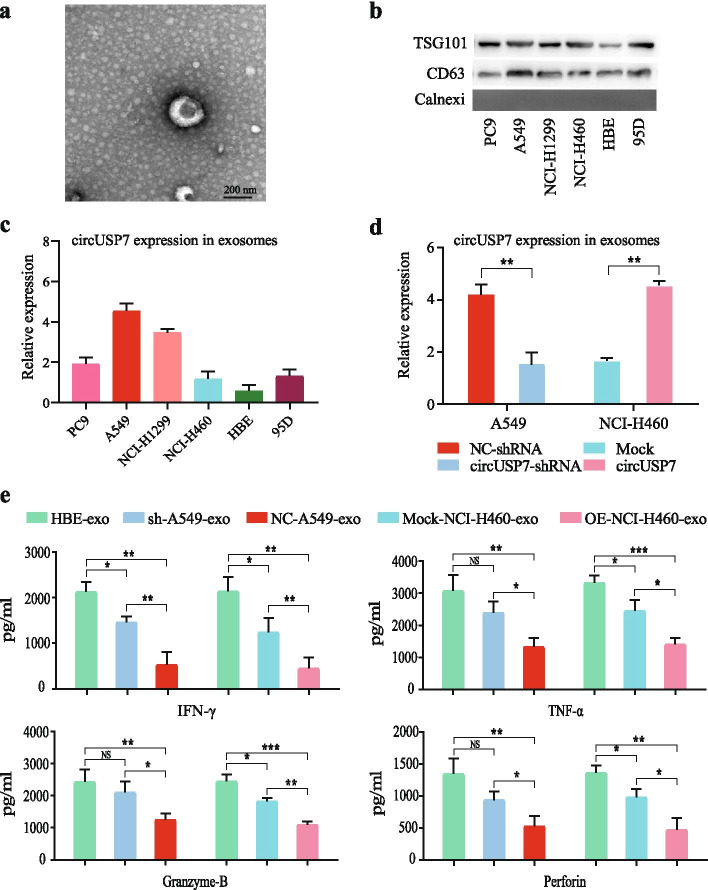
Characterization of exosomes and inhibition of CD8+ T cell function by circUSP7 expression. **a** High-resolution TEM image of NSCLC cell-derived exosomes (scale bar = 100 nm). **b** The expression of exosomal biomarkers in NSCLC cell-derived exosomes was detected by western blot. **c** CircUSP7 expression in NSCLC cell-derived exosomes was measured by qRT-PCR. **d** CircUSP7 expression in plasma exosomes derived from A549 cells was modified by transfection with shRNA to knock down its expression, and circUSP7 expression in plasma exosomes derived from NCI-H460 cells was modified by transfection with cDNA to increase its expression. **e** The secretion of IFN-γ, TNF-α, Perforin, and Granzyme-B by CD8^+^ T cells cocultured with exosomes derived from A549 cells transfected with shRNA or exosomes derived from NCI-H460 cells transfected with cDNA was analysed by ELISA. The data are presented as the mean ± SD. **P < 0.01. NS: not significant

Previous results proved the existence of circUSP7 in the peripheral blood plasma exosomes of NSCLC patients, and the expression of plasma exosome circUSP7 was shown to have a clear relationship with the number of CD8^+^ T cells. Recently, an increasing number of studies have reported that NSCLC cell-derived exosomes participate in cancer progression. Therefore, we identified exosomes from the supernatants of cultured NSCLC cell lines and assessed these exosomes by TEM and western blotting (Fig. 3a and b). Interestingly, qRT-PCR analysis of circUSP7 expression in the NSCLC cell lines and the corresponding exosomes showed similar trends, and the results showed that the circUSP7 levels in the A549 and NCI-H1299 cell lines were higher than those in the other cell lines. Furthermore, the circUSP7 levels in the NCI-H460 and 95D cell lines were decreased compared with those in the other NSCLC cell lines, similar to the expression of exosomal circUSP7 in the supernatants of the NSCLC cell lines (Fig. 3c and Additional File 2: Supplementary Fig. 2). Next, we overexpressed circUSP7 in the NCI-H460 and 95D cell lines. In addition, shRNAs targeting the back splicing site were transfected into A549 and NCI-H1299 cells to knockdown circUSP7 expression (Fig. 3c and Additional File 2: Supplementary Fig. 3), Importantly, the expression levels of e xosomal circUSP7 in the cell supernatants of the circUSP7 overexpression group were increased compared with those in the cell supernatants of the control group (NCI-H460 and 95D cells), and the expression levels of exosomal circUSP7 in the cell supernatants of the circUSP7 knockdown group were decreased compared with those in the cell supernatants of the NC group (A549 and NCI-H1299) (Fig. 3d and Additional File 2: Supplementary Fig. 3). To determine the function of exosomal circUSP7 during tumour immune evasion, we isolated exosomes from the culture media of NSCLC cell lines or the normal human bronchial epithelial cell line HBE, incubated these exosomes with CD8^+^ T cells for 48 h, and then activated the T cells with CD3 antibodies. The results showed that CD8^+^ T cells incubated with A549 cell-derived exosomes secreted significantly lower amounts of TNF-α, IFN-γ, Granzyme-B, and perforin than CD8^+^ T cells incubated with HBE cell- and NCI-H460 cell-derived exosomes (Fig. 3e and Additional File 2: Supplementary Fig. 4); these results indicated that exosomal circUSP7 inhibits CD8^+^ T cell secretion of cytokines, including TNF-α, IFN-γ, Granzyme-B and perforin, which inhibit tumour cell growth and ultimately lead to immune escape of NSCLC.

### CircUSP7 inhibits the biological function of miR-934 in CD8^+^ T cells

**Fig. 4:**
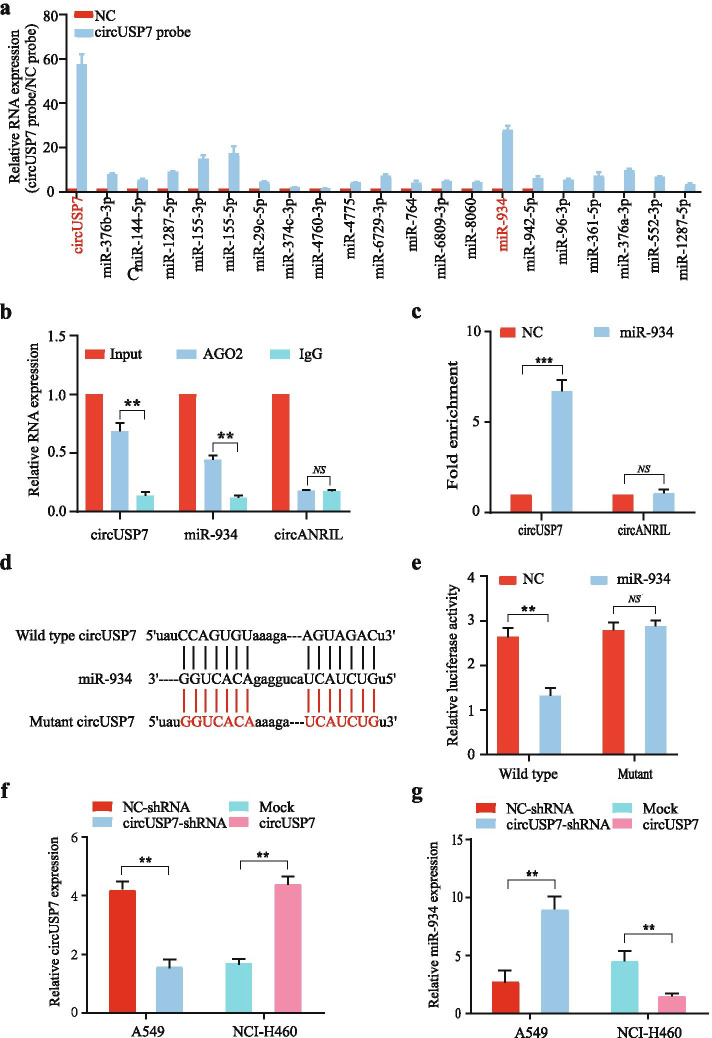
CircUSP7 inhibits the biological function of miR-934 in CD8+ T cells. **a** RIP was performed for circRNA in CD8^+^ T cells using a circUSP7 probe and a NC probe. **b** RIP experiments were carried out in CD8^+^ T cell extracts using an anti-AGO_2_ antibody. **c** The level of circUSP7 in the streptavidin-captured fractions from CD8^+^ T cell lysates after transfection with biotinylated miR-934 or the NC. CircANRIL was used as the negative control. **d** Putative binding sites of miR-934 in the circUSP7 sequence were predicted by StarBase v3.0. **e** The luciferase activity of pGL3-circUSP7 in CD8^+^ T cells after cotransfection with miR-934. **f** CircUSP7 expression in CD8^+^ T cells cocultured with exosomes derived from A549 cells and NCI-H460 cells. **g** The relative level of miR-934 in CD8^+^ T cells transfected with NC, shcircUSP7, mock, or circUSP7 was measured by qRT-PCR. The data are presented as the mean ± SD; **P < 0.01, ***P < 0.001

The functions and characteristics of circRNAs have been reported in many kinds of studies. In addition to some circRNAs that can specifically translate proteins, circRNAs mainly act as sponges of miRNAs and thus affect the biological functions of the miRNAs to which they bind. To verify the hypothesis that a corresponding miRNA binds to circUSP7 in CD8^+^ T cells, we used StarBase v 3.0, circBank and circInteractome analyses to predict whether 20 candidate miRNAs bind to circUSP7. To facilitate the search for miRNAs that bind to circUSP7 in human CD8^+^ T cells, specific probes were used to assess the binding of 20 candidate miRNAs to circUSP7 through circRIP. The data showed that miR-934 was significantly enriched in the circUSP7 group compared with the control group, but other miRNAs were not significantly different in the circUSP7 group compared with the control group, which indicated that miR-934 might interact with circUSP7 in CD8^+^ T cells (Fig. 4a). Next, RIP assays with an AGO2 antibody were conducted in CD8^+^ T cells to confirm whether circUSP7 acts as a miR-934 sponge in these cells, and the results showed that circUSP7 and miR-934, but not circANRIL (a circRNA reported not to bind to AGO2) [34, 35], were enriched (Fig. 4b). To further determine the binding relationship between circUSP7 and miR-934, an RNA pulldown experiment was carried out in CD8^+^ T cells. As expected, the results showed that circUSP7 was significantly enriched in CD8^+^ T cells in the miR-934 group (Fig. 4c) compared to those in the NC group. To further verify this result, a pGL3 luciferase plasmid containing either the wild-type circUSP7 sequence or a miR-934 binding site-mutant circUSP7 sequence was cotransfected with miR-934 mimics into CD8^+^ T cells, and the results showed that the luciferase activity of the cells transfected with the wild-type circUSP7 sequence was significantly decreased compared with that of the cells transfected with the miR-934 binding site-mutant sequence (Fig. 4d and e). This result clearly confirmed our prediction results described above. Moreover, as the level of circUSP7 increased, the level of miR-934 decreased, and the level of circUSP7 was significantly decreased after miR-934 was overexpressed in CD8^+^ T cells (Fig. 4f and g; Additional File 2: Supplementary Fig. 5a and 5b). Therefore, we hypothesized that circUSP7 may inhibit miR-934 activity to promote the functional impairment of CD8^+^ T cells. To test this hypothesis, we ectopically modified exosomes derived from NSCLC cells by the transfection of circUSP7 shRNA and circUSP7 cDNA together with miR-934 into CD8^+^ T cells to evaluate the mechanism by which circUSP7 regulates miR-934 (Additional File 2: Supplementary Fig. 6a and 6b). These findings suggested that circUSP7 and miR-934 may target each other in CD8^+^ T cells.

We hypothesized that miR-934 activity may be inhibited by circUSP7 to promote the functional impairment of CD8^+^ T cells. To verify this hypothesis, we detected the secretion of TNF-α, IFN-γ, Granzyme-B, and perforin from CD8^+^ T cells after coculturing CD8^+^ T cells with exosomes derived from NSCLC cells (Additional File 2: Supplementary Fig. 6c and 6d). The data suggested that circUSP7 and miR-934 may affect each other in CD8^+^ T cells and lead to the immune escape of NSCLC.

### CircUSP7 promotes SHP2 expression by inhibiting miR-934 in CD8^+^ T cells

**Fig. 5:**
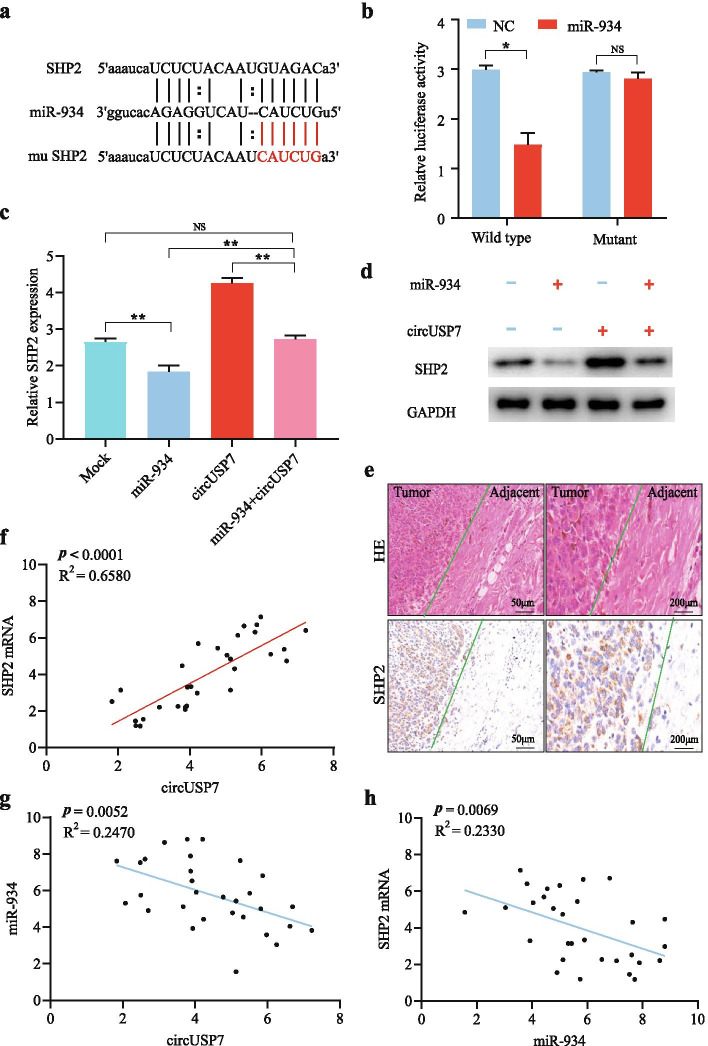
CircUSP7 regulates the miR-934/SHP2 pathway in CD8^+^ T cells. **a** The putative binding site of miR-934 in the SHP2 sequence was predicted by StarBase v3.0. **b** Luciferase activity of pGL3-SHP2 in CD8^+^ T cells cotransfected with miR-934. **c** and **d** The mRNA and protein levels of SHP2 were measured by qRT-PCR and western blotting, respectively, in CD8^+^ T cells transfected with miR-934 or miR-934 combined with circUSP7. **e** SHP2 in representative NSCLC patients was analysed by IHC staining. **f** A positive correlation between circUSP7 and SHP2 mRNA expression was observed in the CD8^+^ T cells from NSCLC patients (R2 = 0.6580; *P* < 0.0001). **g** A negative correlation between circUSP7 and miR-934 expression was observed in the CD8+ T cells from NSCLC patients (R2 = 0.2470; *P* = 0.0052). **h** A negative correlation between miR-934 and SHP2 mRNA expression was observed in the CD8^+^ T cells from NSCLC patients (R^2^ = 0.2330; *P* = 0.0069). The data are presented as the mean ± SD; *P < 0.05, **P < 0.01, NS: not significant

The experiments described above proved that circUSP7 can inhibit the function of CD8^+^ T cells and lead to the immune escape of NSCLC by interacting with miR-934. We hypothesized that circUSP7 may achieve CD8^+^ T cell inhibition by downregulating the expression of the miR-934 target by serving as a miR-934 sponge and that circUSP7 may thus increase the immune evasion of NSCLC in patients. To determine whether the expression of the miR-934 target is increased due to circUSP7 inhibition, we measured the expression level of miR-934 in CD8^+^ T cells. We predicted that SHP2 may be a target mRNA of miR-934 in CD8^+^ T cells based on several bioinformatics analyses, including analyses with the StarBase v 3.0, PITA, and miRanda algorithms (Fig. 5a). The pLG3 luciferase plasmid, which was designed to depend on the firefly luciferase-expressing wild-type SHP2 3′ UTR sequence or a SHP2 3′ UTR sequence with a mutated miR-934-binding site, was used to conduct the luciferase reporter assay. Next, the pGL3 luciferase plasmid and NC or miR-934 mimic were cotransfected into CD8^+^ T cells, and the luciferase activities were analysed. The results demonstrated that compared with the NC, the miR-934 mimics significantly diminished the luciferase reporter activity of the wild-type SHP2 sequence (Fig. 5b). In conclusion, the expression level of SHP2 was suppressed by the overexpression of miR-934.

To further elucidate the function of SHP2-related pathways in the immune evasion of NSCLC in patients, previous studies have suggested that circRNAs play a crucial role in cancer via their strong circRNA activity. The results of a previous experiment suggested that SHP2 mRNA is specifically targeted by miR-934 in CD8^+^ T cells. Hence, we tried to analyse the mechanism by which circUSP7 and miR-934 affect the expression level of SHP2. We detected SHP2 expression in CD8^+^ T cells transfected with plasmids overexpressing exosomal circUSP7 alone, miR-934 mimics alone or both. The results showed that circUSP7 overexpression alone contributed to increased mRNA and protein expression levels of SHP2; in contrast, the miR-934 mimics could reverse the effect of circUSP7 on facilitating SHP2 expression (Fig. 5c and d). In addition, the expression levels of SHP2 in NSCLC adjacent tissues were lower than those in tumour tissues (Fig. 5e), as predicted.

Here, CD8^+^ T cells isolated from the peripheral blood of 30 NSCLC patients were used to further confirm the conclusion that circUSP7 influenced CD8^+^ T cell activity by regulating the miR-934/SHP2 axis. We found that circUSP7 expression was positively associated with SHP2 expression in peripheral blood CD8^+^ T cells (R^2^ = 0.6580; *P* < 0.0001; Fig. 5f). Moreover, circUSP7 expression was negatively related to miR-934 expression in peripheral blood CD8^+^ T cells (R^2^ = 0.2470; *P* = 0.0052; Fig. 5g), as expected. Strikingly, we found that miR-934 expression was negatively associated with SHP2 expression in peripheral blood CD8^+^ T cells (R^2^ = 0.2330; *P* = 0.0069; Fig. 5h), indicating that the function of miR-934 in inhibiting tumour progression by acting on SHP2 was reversed by increasing the levels of exosomal circUSP7 in CD8^+^ T cells.

### CircUSP7 promotes NSCLC progression in a CD8^+^ T cell-dependent manner and leads to resistance to anti-PD1 therapy

**Fig. 6:**
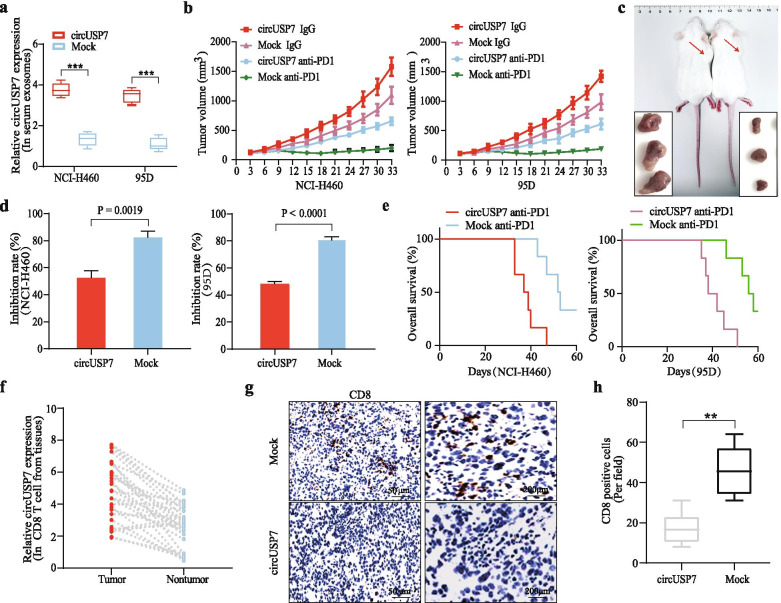
CircUSP7 promotes NSCLC progression in a CD8^+^ T cell-dependent manner and leads to resistance to anti-PD1 therapy. **a** The relative expression levels of circUSP7 in the serum exosomes from HuNSG mice. **b** and **c** The data are expressed as the mean tumour volume (the data are presented as the mean ± SD; *n* = 6). **d** The data are expressed as the percent of tumours with inhibited growth (the data are presented as the mean ± SD; *n* = 6). **e** Comparison of the OS curves for mice with xenograft NSCLC tumours with high and low circUSP7 expression that were treated with Opdivo. **f** The expression of circUSP7 in the CD8^+^ T cells from NSCLC patient tissues (*n* = 30). **g** CD8-positive cells in NCI-H460-circUSP7 or NCI-H460-mock cell-derived tissues were analysed by IHC. The data are presented as the mean ± SD; *P < 0.05, **P < 0.01

To further analyse the mechanism by which circUSP7 promotes tumour progression in NSCLC, first, a HuNSG mouse model of a humanized immune system (mice were injected with hCD34^+^-purified HPSCs derived from healthy donors) was constructed to verify the function of circUSP7 in NSCLC. To further observe and verify the role of the circUSP7/miR-934/SHP2 axis in vivo, we showed that the circUSP7 level in the peripheral blood exosomes from HuNSG mice administered NCI-H460 and 95D cells transfected with the pLO5-ciR-circUSP7 overexpression vector was significantly higher than that in the peripheral blood exosomes from HuNSG mice administered cells transfected with the pLO5-ciR-Mock vector (Fig. 6a). The results indicated that NSCLC cells can secrete exosomes and that circUSP7 is indeed present in the peripheral blood of HuNSG mice. To verify the effect of circUSP7 on resistance to PD1 monoclonal antibodies, we analysed the effect of PD1 antibodies on HuNSG mice that received circUSP7-overexpressing cells (including NCI-H460 and 95D) or the respective mock cells. Compared to the mock mice with low exosomal circUSP7 expression, the xenograft mice with high exosomal circUSP7 expression showed an obvious phenotype of resistance to anti-PD1 treatment, and the xenograft mice had a shorter survival time (Fig. 6b-e).

Next, we isolated CD8^+^ T cells from the cancer and adjacent tissues of 30 NSCLC patients and detected the expression of circUSP7 in these CD8^+^ T cells. The results showed that the expression level of circUSP7 in CD8^+^ T cells from cancer tissues was significantly higher than that in the corresponding CD8^+^ T cells from adjacent tissues (Fig. 6f). In addition, the number of CD8^+^ T cells in the metastatic tumour nodules induced by the mock-transfected NSCLC cells was significantly increased compared with that in the tumour nodules induced by the circUSP7-transfected NSCLC cells (Fig. 6g and h). These results indicated that circUSP7 promotes NSCLC progression and PD1 treatment resistance in an exosome- and CD8^+^ T cell-dependent manner.

### Upregulation of circUSP7 induces T lymphocyte exhaustion by the miR-934/SHP2 axis in NSCLC patients

**Fig. 7:**
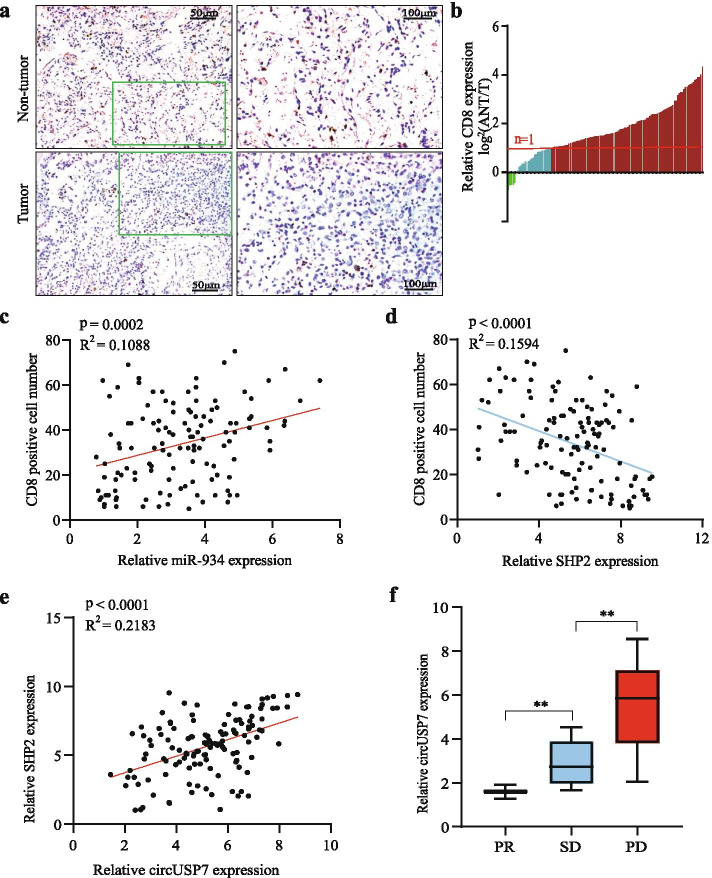
Upregulation of CircUSP7 induces cytotoxic T lymphocyte exhaustion by interacting with the miR-934/SHP2 axis in NSCLC patients. **a** CD8 in tissues from representative NSCLC patients was analysed by IHC staining. **b** CD8^+^ T cells in 126 pairs of NSCLC tissues and matched nontumour tissues, shown as log2 (tumour/nontumour). **c** A positive correlation between circUSP7 expression and CD8-positive cell number was observed in the NSCLC tissues (R^2^ = 0.1088; *P* = 0.0002). **d** A negative correlation between SHP2 expression and CD8-positive cell number was observed in the NSCLC tissues (R^2^ = 0.1594; *P* < 0.0001). **e** A positive correlation between circUSP7 and SHP2 mRNA was observed in NSCLC tissues (R^2^ = 0.2183; *P* < 0.0001). **f** The expression level of circUSP7 in the plasma exosomes of these patients after anti-PD1 treatment cycles. The data are presented as the mean ± SD; *P < 0.05, **P < 0.01

We have demonstrated that circUSP7 promotes NSCLC progression and anti-PD1 immunotherapy resistance in vitro and in vivo. To further explore the correlation between the circUSP7/SHP2 axis and immune escape in NSCLC patients, we measured the infiltration of CD8^+^ T cells into cancer tissues and matched nontumour tissues from 126 NSCLC patients. The number of CD8^+^ T cells in the NSCLC tissues was significantly lower than that in the adjacent nontumour tissues (Fig. 7a and b). The results from a scatter plot analysis revealed a negative relationship between miR-934/SHP2 expression and CD8^+^ T cell frequency in NSCLC tissues (Fig. 7c and d). These results revealed that upregulation of circUSP7 expression promotes SHP2 expression and may exert its immunosuppressive effects by inhibiting CD8^+^ T cell function. Then, we observed a positive correlation between circUSP7 and SHP2 in NSCLC patient tissue samples (Fig. 7e). In addition, using qRT-PCR, we measured the expression of miR-934 in the tumour tissues of 126 NSCLC patients. A negative relationship between circUSP7 and miR-934 was observed in NSCLC patient tissues (Additional File 2: Supplementary Fig. 7a and 7b). We next evaluated whether circUSP7 overexpression can further inhibit the antitumour effect of anti-PD1 therapy (Opdivo) in NSCLC patients. Thus, we analysed 20 NSCLC patients who received anti-PD1 immunotherapy after six treatment cycles, and CT was used to evaluate the efficacy based on RECIST1.1 analysis. According to RECIST1.1, patients are categorized as having progressive disease (PD), stable disease (SD), partial response (PR), and complete response (CR). The results showed that there were 2 patients with a PR, 6 patients with SD, and 12 patients with PD (Additional File 2: Supplementary Fig. 7c). We continued to detect the expression level of circUSP7 in the plasma exosomes of these patients after anti-PD-1 treatment cycles. As expected, the expression level of circUSP7 in the PD group was significantly higher than that in the PR and SD groups (Fig. 7f; Additional File 2: Supplementary Fig. 7d and 7e). Therefore, circUSP7 induces cytotoxic T lymphocyte exhaustion in vivo and in vitro.

## Discussion

NSCLC, a global public health issue, remains one of the most common cancers worldwide [36]. The lack of effective interventions and specific molecular targets are the principal reasons for the failure of NSCLC treatment; consequently, it is significant to search for novel molecular therapeutic targets for NSCLC.

Recently, an increasing number of studies have suggested that circRNAs are abnormally expressed in various cancers and have shown that many circRNAs play important roles in regulating tumour immunosuppression, migration, invasion, metastasis, proliferation, and chemotherapy resistance. However, the biological molecular mechanisms of circRNAs in multiple tumour types are still unclear. Therefore, we proved that circUSP7, which is derived from USP7, can promote NSCLC progression and assessed the biological molecular mechanisms by which it enhances NSCLC immunosuppression. First, we found that circUSP7 was highly expressed in NSCLC tissues and NSCLC-derived exosomes. Furthermore, we proved that exosomal circUSP7 secreted from NSCLC tissues was transferred into CD8^+^ T cells and led to tumour immunosuppression, upregulated SHP2 expression by sponging miR-934, and, in turn, caused CD8^+^ T cell exhaustion. In particular, we found that NSCLC cells with high circUSP7 expression were resistant to anti-PD1 treatment in HuNSG mice with a humanized immune system. All the results described above indicated that exosomal circUSP7 secreted by NSCLC cells mediates resistance to anti-PD1 therapy by inducing CD8^+^ T cell exhaustion, which may provide a previously undescribed therapeutic target for the treatment of NSCLC patients.

An increasing number of studies have found that exosomes are enriched in circRNAs, which may participate in the transmission of information and communication between tumour cells [23, 37, 38]. In addition, in many diseases, exosomes facilitate progression to altered cellular or tissue states, and their discovery in biological fluids potentially offers a multicomponent diagnostic readout. The effective exchange of cellular components through exosomes can inform their use in the design of exosome-based treatment methods. Here, our study emphasized a vital role for exosomal circUSP7 in promoting immune evasion and resistance to anti-PD1 therapy by inducing CD8^+^ T cell dysfunction in NSCLC.

Increasingly, a large number of studies have reported that plasma exosomal circRNAs are present in the peripheral blood of patients with many cancers, including NSCLC [27, 39–42]. More importantly, circRNAs have been verified to be involved in the regulation of the response of a variety of cancers to the immune system, including the immune evasion of cancer [43]. Our results demonstrate that circUSP7 promotes tumour progression and plays a role in immune evasion in NSCLC patients. Patients with high levels of exosomal circUSP7 will be resistant to anti-PD1 therapy. Moreover, we further showed that the levels of exosomal circUSP7 were not related to *TP53, KRAS* or ALK mutations, which have been identified as potential predictive factors in guiding anti-PD1/PD-L1 immunotherapy [44] (Additional File 2: Supplementary Fig. 8). Thus, we conclude that the level of exosomal circUSP7 can serve as a newly predicted marker of anti-PD1 therapy in NSCLC.

The prognosis of patients with a variety of cancers is affected by their CD8^+^ T cell numbers. PD1 is expressed by activated lymphocytes, including T cells, NK cells, and B cells, and acts as an inhibitory receptor [45, 46]. High levels of PD1 expression on CD8^+^ T cells inhibit the functional regulation of these activated cells [47]. A large amount of research has revealed that anti-PD1 therapy promotes CD8^+^ T cell activation to enhance immunity [48]. SHP2 is a crucial coinhibitory molecule expressed on T cells. SHP2 was expressed at high levels in the peripheral blood CD8^+^ T cells of cancer patients and mediated the immunosuppressive effects of these cells [8, 49, 50]. Furthermore, increased SHP2 expression is related to immune dysfunction and CD8^+^ T cell exhaustion [50]. Previous studies have also reported that allosteric inhibitors of SHP2 augment antitumour immunity and elevate the proportion of CD8^+^ T cells [10, 51]. Our results in this study showed that the expression of circUSP7 was augmented not only in NSCLC tissues but also in NSCLC-derived exosomes. Importantly, poor prognosis and pathological characteristics were associated with high expression of circUSP7 and led to tumour-promoting effects on NSCLC. In addition, the higher expression of exosomal circUSP7 derived from NSCLC cells inhibits the capability of CD8^+^ T cells to produce TNF-α, IFN-γ, Granzyme-B, and perforin by upregulating SHP2 expression and promoting CD8^+^ T cell exhaustion.

PD1 is a negative costimulatory receptor critical for the suppression of T cell activation in vitro and in vivo and is associated with SHP2 [8, 10, 52]. Furthermore, SHP2 plays a central and indispensable role in oncogenic KRAS-driven tumours and promotes tumour development [53]. Here, we found that increased circUSP7 expression reduced the therapeutic efficacy of anti-PD1 treatment via the exosomal circUSP7/miR-934/SHP2 axis. Thus, we revealed that circUSP7 levels are a crucial factor that affects resistance to anti-PD1 therapy in NSCLC patients, and peripheral blood exosomal circRNAs can be used as tumour markers. Importantly, an inhibitor of SHP2 may decrease the immunosuppressive effects observed in patients with high circUSP7 expression by inhibiting the immune function of CD8^+^ T cells.

## Conclusion

PD1 is a negative costimulatory receptor critical for the suppression of T cell activation in vitro and in vivo and is associated with SHP2 [8, 10, 52]. Furthermore, SHP2 plays a central and indispensable role in oncogenic KRAS-driven tumours and promotes tumour development [53]. Here, we found that increased circUSP7 expression reduced the therapeutic efficacy of anti-PD1 treatment via the exosomal circUSP7/miR-934/SHP2 axis. Thus, we revealed that circUSP7 levels are a crucial factor affecting resistance to anti-PD1 therapy in NSCLC patients, and peripheral blood exosomal circRNAs can be used as a tumour marker. Importantly, an inhibitor of SHP2 may ameliorate the immunosuppressive effects caused by inhibition of CD8^+^ T cells in patients with higher circUSP7 expression.

## Supplementary Information

**Additional file 1: Supplementary Table 1.** Data of sequences for qPCR and shRNA in this study. **Supplementary Table 2.** Antibody for western blot, RIP, and immunohistochemistry.

**Additional file 2: Supplementary Figure 1.** SHP2 and miR-934 expression along with cirUSP7 in NSCLC tissues were measured using RT-qPCR analysis according to groups include lymph node metastasis, tumor size, and intravascular cancer embolus. **Supplementary Figure 2.** circUSP7 expression in several NSCLC cell lines was measured using RT-qPCR analysis. **Supplementary Figure 3.** The expression of circUSP7 in NSCLC cell lines and its exosomes. a circUSP7 expression in the A549 and NCI-H1299 cells was modified by transfection of shRNA to cause interference, and circUSP7 expression in the NCI-H460 and 95D cells were modified by cDNA transfection. b circUSP7 silencing is accompanied by decreased exosomal circUSP7 in A549 and NCI-H1299 cells, and circUSP7 overexpression is accompanied by increased exosomal circUSP7 in NCI-H460 and 95D cells. The data are presented as the mean ± SD. **P < 0.01. **Supplementary Figure 4.** The secretion of IFN-γ, TNF-α, Perforin, and Granzyme-B expression in CD8 + T cells co-cultured with exosomes derived from NCI-H1299 cells was modified by transfection of shRNA and 95D cells was modified by cDNA transfection by ELISA. **Supplementary Figure 5.** The relationship between circUSP7 and miR-934 expression in CD8 + T cells. a circUSP7 or miR-934 expression in CD8^+^T cells was co-cultured with exosome derived from NCI-H1299 cells was modified by transfection of shRNA and 95D cells were modified by cDNA transfection. b circUSP7 expression in CD8^+^T cells was modified by miR-934 mimics or shRNA transfection, miR-934 expression in miR-934-overexpressing or miR-934-silenced CD8^+^T cells. The data are presented as the mean ± SD. **P < 0.01. **Supplementary Figure 6.** miR-934 affects the function of circUSP7 in CD8^+^T cells. a The expression of circUSP7 and miR-934 in CD8^+^T cells co-cultured with exosomes derived from NCI-H460 and 95D cells was modified by transfection of shRNA, and modified with miR-934 expression. b The expression of circUSP7 and miR-934 in CD8^+^T cells co-cultured with exosomes derived from NCI-H1299 and A549 cells was modified by transfection of cDNA, and modified with miR-934 expression. **c** The secretion of IFN-γ, TNF-α, Perforin, and Granzyme-B expression in CD8 + T cells co-cultured with exosomes derived from NCI-H460 cells was modified by cDNA transfection with modified circUSP7 and miR-934 expression. d The secretion of IFN-γ, TNF-α, Perforin, and Granzyme-B expression in CD8^+^T cells co-cultured with exosomes derived from A549 cells was modified by transfection of shRNA with modified circUSP7 and miR-934 expression. **Supplementary Figure 7.** a A negative correlation between circUSP7 and miR-934 was observed in the NSCLC tissues (R^2^ = 0.1282; *P* < 0.0001). b A negative correlation between SHP2 mRNA and miR-934 was observed in the NSCLC tissues (R^2^ = 0.2258; *P* < 0.0001). c The PD1 antibody immunotherapy efficacy assessment using CT-based RECIST1.1. d and e The relationship between circUSP7 and miR-934, SHP2 and circUSP7, and SHP2 and miR-934 expression in patients with PD or PR + SD after anti-PD1 therapy. **Supplementary Figure 8.** The relationship between circUSP7 and Kras, p53 and ALK mutation status in NSCLC.

## Data Availability

All data generated or analysed during this study are included either in this article or in the supplementary information files.

## Abbreviations

NSCLC: Non-small cell lung cancer
OS: Overall survival
RFS: Recurrence-free survival
SHP2: qRT-PCR real-time quantitative polymerase chain reaction
TMAs: Tissue microarrays
Co-IP: Coimmunoprecipitation
RIP: RNA immunoprecipitation
AGO2: Argonaute 2
PD-1: Programmed cell death 1
USP7: Ubiquitin-specific protease-7
SHP2: Cytoplasmic Src homology 2 (SH2) domain-containing phosphatase 2
PTPN11: Protein tyrosine phosphatase nonreceptor type 11
IHC: Immunohistochemistry
DMEM: Dulbecco’s modified Eagle medium
