# Supplementary material for: Cancer cell-derived exosomal circUSP7 induces CD8+ T cell dysfunction and anti-PD1 resistance by regulating the miR-934/SHP2 axis in NSCLC
Source: Mol Cancer. 2021 Nov 9;20:144. doi: 10.1186/s12943-021-01448-x (PMC8576933; doi:10.1186/s12943-021-01448-x)
Supplement: Supplementary file 1 — Additional file 1: Supplementary Table 1. Data of sequences for qPCR and shRNA in this study. Supplementary Table 2. Antibody for western blot, RIP, and immunohistochemistry. [file 12943_2021_1448_MOESM1_ESM.docx]

**Supplementary Table 1:** Data of sequences for qPCR and shRNA in this study

| **RNA or Gene** | **Sequence** |
| --- | --- |
| hsa_circ_0106173 | Forward: AGTCATTTGGGTGGGATTCA  Reverse: GCCTTTCGTAGCTGATTCGT |
| hsa_circ_0106168 | Forward: AAAATGGTGTCCTATATCCAGTGT  Reverse: CGGTGTTGTGTCCATCACTC |
| hsa_circ_0106167 | Forward: CATTTGTGGATTATGTGGCAGT  Reverse: AAGGGACGCTTTTAGACGAA |
| hsa_circ_0106164 | Forward: AAATTCCTAACATTGCCACCA  Reverse: AAGGGACGCTTTTAGACGAA |
| hsa_circ_0106153 | Forward: AGCACAATTATGGGGGTCAC  Reverse: AAAGGGACGCTTTTAGACGA |
| hsa_circ_0106152 | Forward: AGCACAATTATGGGGGTCAC  Reverse: CGAGCTGTTCTACTGCCACA |
| hsa_circ_0106151 | Forward: CAGTTGGTGGAGCGATTACA  Reverse: GTTCCCCAGCGTCGTATTTA |
| hsa_circ_0106140 | Forward: CAGGTTGCAAAGACAGTTGC  Reverse: TAATTCAAGCTCCGCGTTTT |
| hsa_circ_0106139 | Forward: GCACAGAGGCTCAACACAGA  Reverse: ATCAACGCGGTGGTAGAGAT |
| hsa_circ_0008638 | Forward: GTGACCGATCCTGAGAAAGG  Reverse: CGGTGTTGTGTCCATCACTC |
| hsa_circ_0005152 | Forward: AAAATGGTGTCCTATATCCAGTG  Reverse: AAGGGACGCTTTTAGACGAA |
| hsa_circ_0004611 | Forward: CGCTTGCTGAGTTTGTTCAG  Reverse: TGAGCCTCGATCCTTTTCTC |
| hsa_circ_0004344 | Forward: CATGAGAAACTGCAGCCGTA  Reverse: CGGTGTTGTGTCCATCACTC |
| miR-934 | Forward: GCCTAGAAACATCCTCCCGG  Reverse: AGGCCATGTGTCGTGGTCG |
| SHP2 | Forward: GGCACAGTACTACAACTCAA  Reverse: TGGTCTCAGCTAATTTGCTT |
| GAPDH | Forward: ACAACTTTGGTATCGTGGAAGG  Reverse: GCCATCACGCCACAGTTTC |
| shhsa_circ_0005152 Target | [AGGAAAGAAAAATAGCTGTGT](http://blast.ncbi.nlm.nih.gov/Blast.cgi?PROGRAM=blastn&PAGE_TYPE=BlastSearch&LINK_LOC=blasthome&QUERY=%3Ehsa_circ_0005152-siRNA1%0AAGGAAAGAAAAATAGCTGTGT&DATABASE=nr&EQ_MENU=Homo%C2%A0sapiens%C2%A0(taxid:9606)) |

**Supplementary Table 2:** Antibody for western blot, RIP, and immunohistochemistry

| **Antibody** | **company** | **Cat NO.** |
| --- | --- | --- |
| SHP2 | Cell Signaling Technology | 3397S |
| GAPDH | Affinity | AF7021 |
| CD8 | Abcam | ab4055 |
| PD1 | Abcam | ab214421 |
| AGO2 | Abcam | ab32381 |
| IgG | Abcam | ab172730 |
| HRP-labeled Anti-Rabbit IgG | Cell Signaling Technology | #7074 |
| HRP-labeled Anti-mouse IgG | Cell Signaling Technology | #75952 |
