# Supplementary material for: Cancer cell-derived exosomal circUSP7 induces CD8+ T cell dysfunction and anti-PD1 resistance by regulating the miR-934/SHP2 axis in NSCLC
Source: Mol Cancer. 2021 Nov 9;20:144. doi: 10.1186/s12943-021-01448-x (PMC8576933; doi:10.1186/s12943-021-01448-x)
Supplement: Supplementary file 2 — Additional file 2: Supplementary Figure 1. SHP2 and miR-934 expression along with cirUSP7 in NSCLC tissues were measured using RT-qPCR analysis according to groups include lymph node metastasis, tumor size, and intravascular cancer embolus. Supplementary Figure 2. circUSP7 expression in several NSCLC cell lines was measured using RT-qPCR analysis. Supplementary Figure 3. The expression of circUSP7 in NSCLC cell lines and its exosomes. a circUSP7 expression in the A549 and NCI-H1299 cells was modified by transfection of shRNA to cause interference, and circUSP7 expression in the NCI-H460 and 95D cells were modified by cDNA transfection. b circUSP7 silencing is accompanied by decreased exosomal circUSP7 in A549 and NCI-H1299 cells, and circUSP7 overexpression is accompanied by increased exosomal circUSP7 in NCI-H460 and 95D cells. The data are presented as the mean ± SD. **P < 0.01. Supplementary Figure 4. The secretion of IFN-γ, TNF-α, Perforin, and Granzyme-B expression in CD8 + T cells co-cultured with exosomes derived from NCI-H1299 cells was modified by transfection of shRNA and 95D cells was modified by cDNA transfection by ELISA. Supplementary Figure 5. The relationship between circUSP7 and miR-934 expression in CD8 + T cells. a circUSP7 or miR-934 expression in CD8+T cells was co-cultured with exosome derived from NCI-H1299 cells was modified by transfection of shRNA and 95D cells were modified by cDNA transfection. b circUSP7 expression in CD8+T cells was modified by miR-934 mimics or shRNA transfection, miR-934 expression in miR-934-overexpressing or miR-934-silenced CD8+T cells. The data are presented as the mean ± SD. **P < 0.01. Supplementary Figure 6. miR-934 affects the function of circUSP7 in CD8+T cells. a The expression of circUSP7 and miR-934 in CD8+T cells co-cultured with exosomes derived from NCI-H460 and 95D cells was modified by transfection of shRNA, and modified with miR-934 expression. b The expression of circUSP7 and miR-934 in CD8+T cells [file 12943_2021_1448_MOESM2_ESM.docx]

**Additional file: Supplementary Figures and Figure legends**

**Supplementary Figure 1.**

**
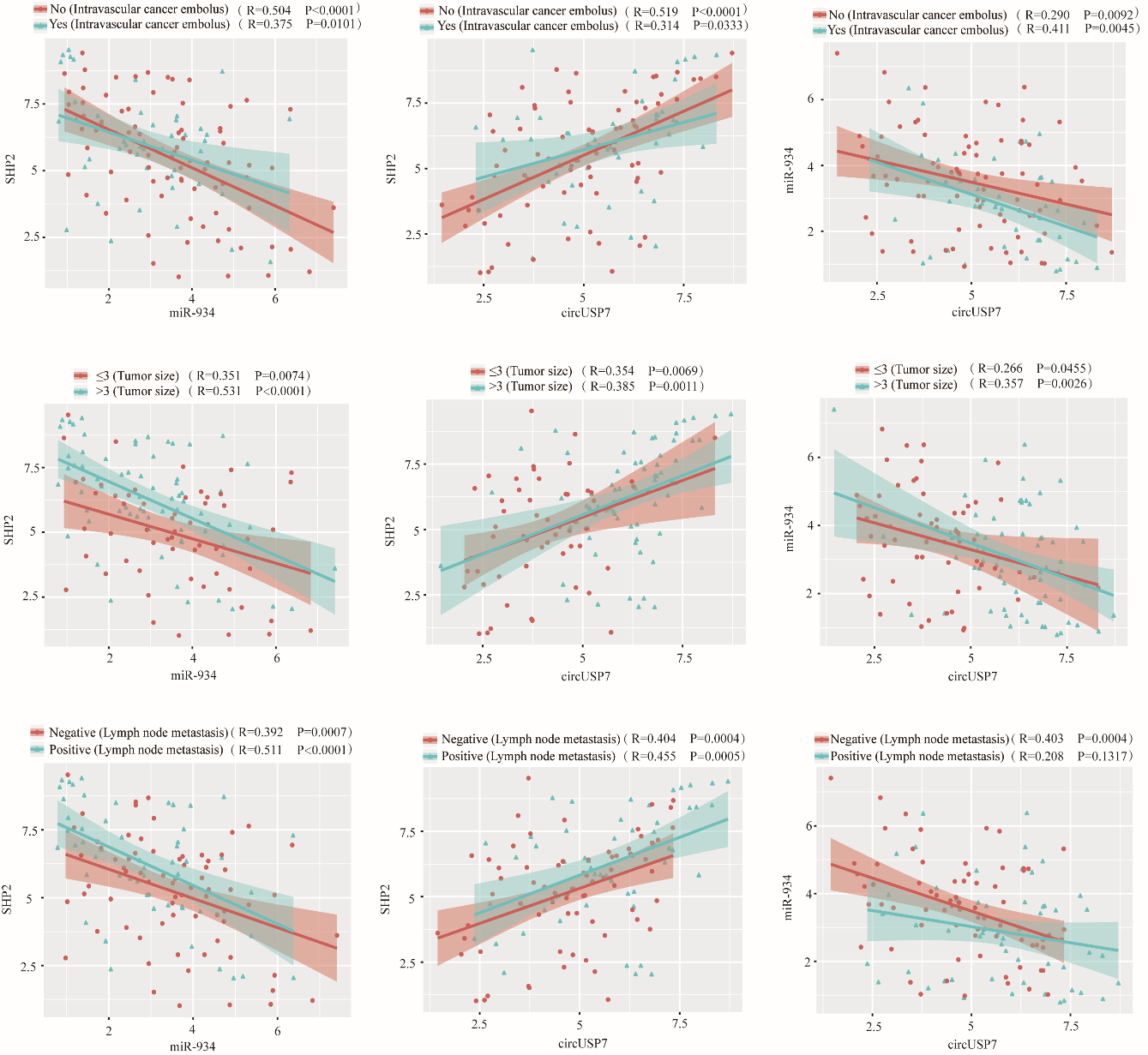
**

**Supplementary Figure. 1.** SHP2 and miR-934 expression along with cirUSP7 in NSCLC tissues were measured using RT-qPCR analysis according to groups include lymph node metastasis, tumor size, and intravascular cancer embolus.

**Supplementary Figure 2.**


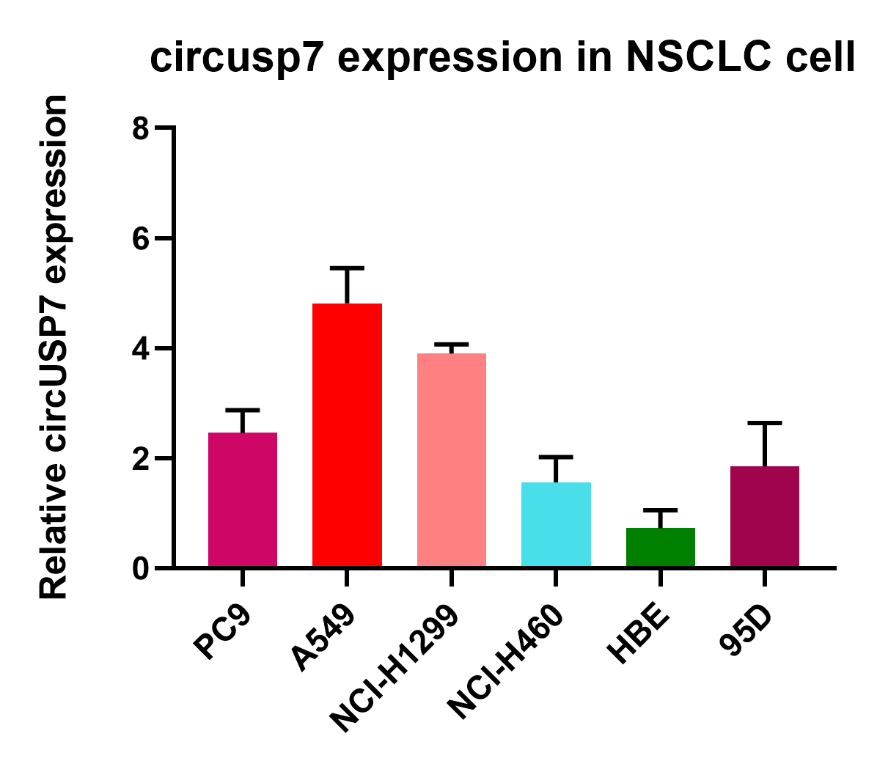


**Supplementary Figure. 2.** circUSP7 expression in several NSCLC cell lines was measured using RT-qPCR analysis.

**Supplementary Figure 3.**


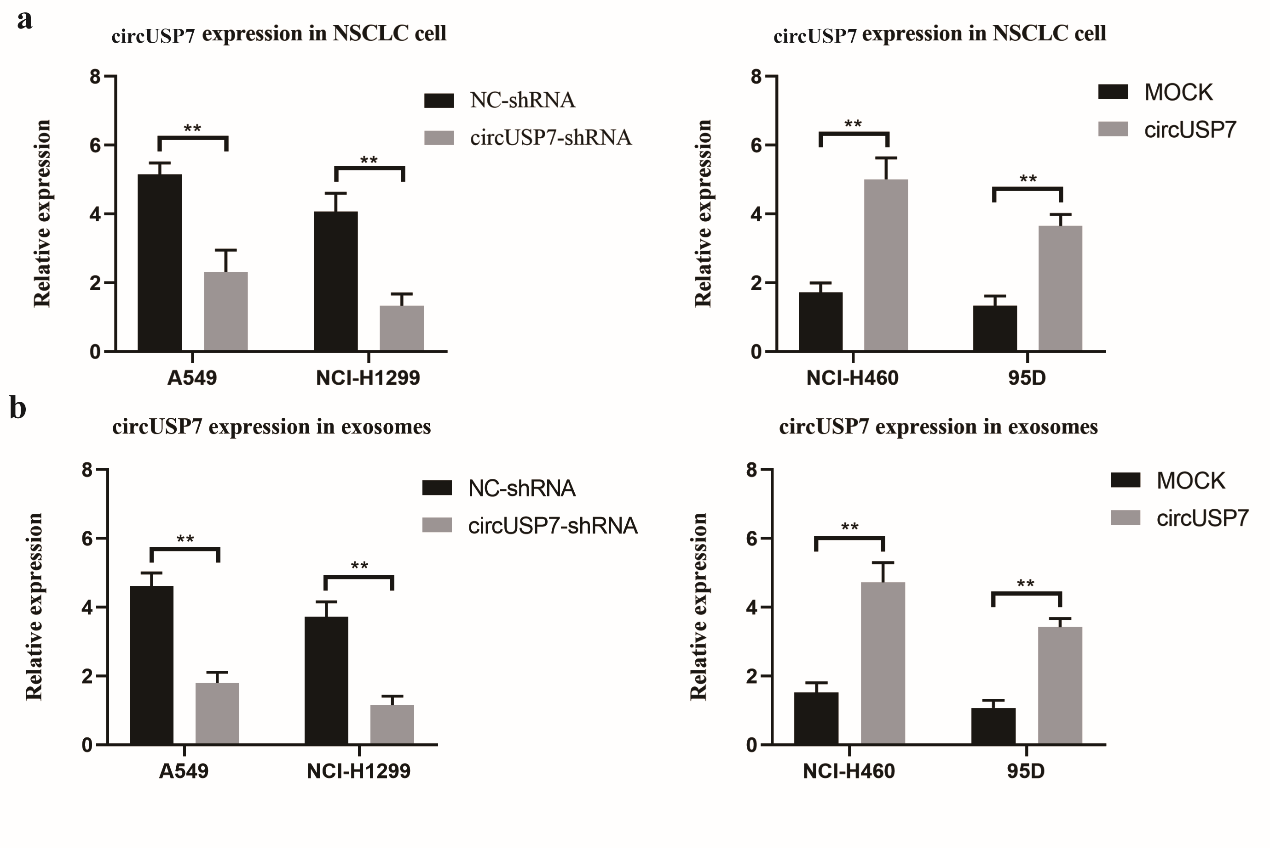


**Supplementary Figure. 3. the expression of circUSP7 in NSCLC cell lines and its exosomes**. **a** circUSP7 expression in the A549 and NCI-H1299 cells was modified by transfection of shRNA to cause interference, and circUSP7 expression in the NCI-H460 and 95D cells were modified by cDNA transfection. **b** circUSP7 silencing is accompanied by decreased exosomal circUSP7 in A549 and NCI-H1299 cells, and circUSP7 overexpression is accompanied by increased exosomal circUSP7 in NCI-H460 and 95D cells. The data are presented as the mean ± SD. **P < 0.01.

**Supplementary Figure 4.**


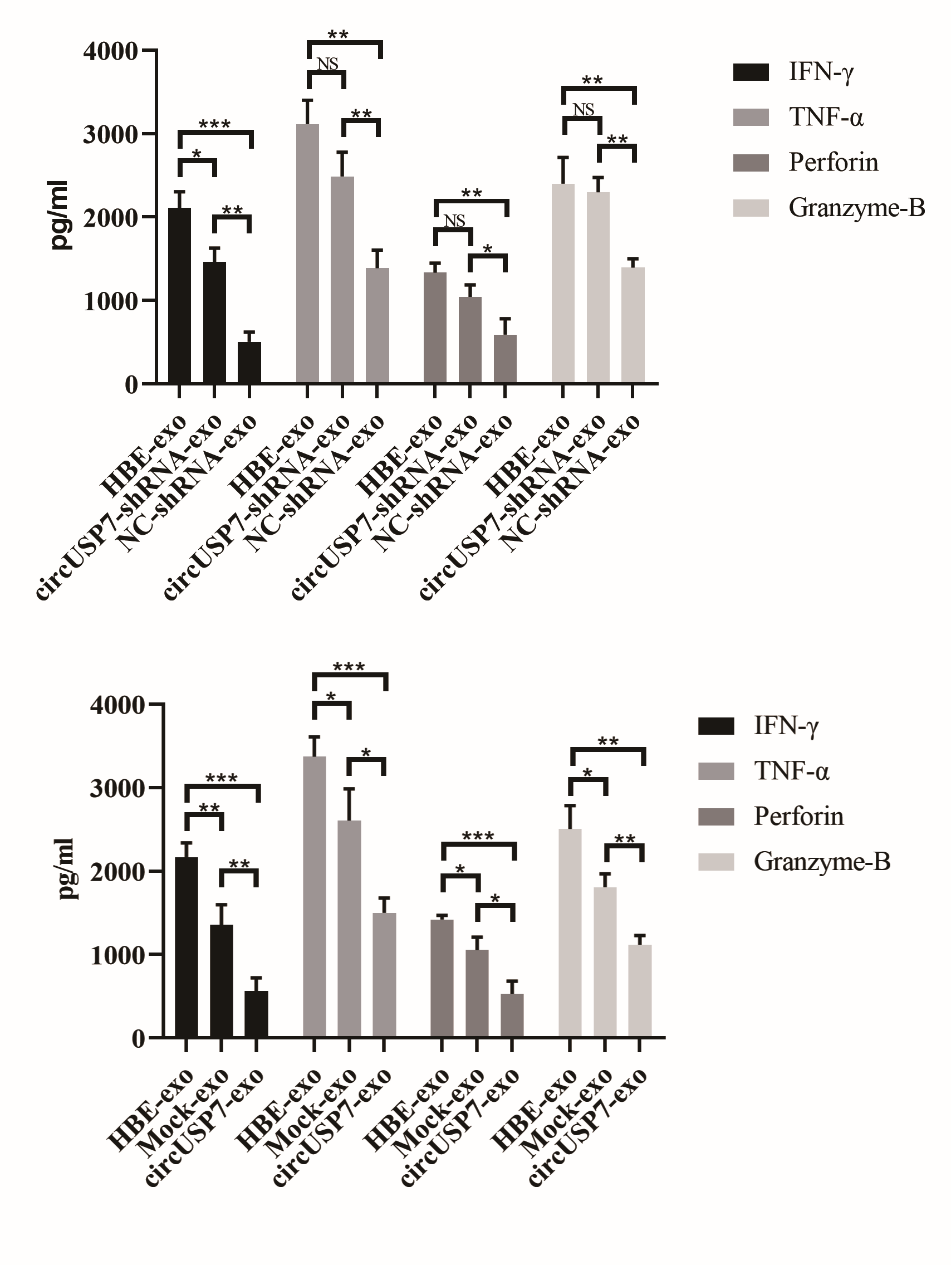


**Supplementary Figure. 4.** The secretion of IFN-γ, TNF-α, Perforin, and Granzyme-B expression in CD8+T cells co-cultured with exosomes derived from NCI-H1299 cells was modified by transfection of shRNA and 95D cells was modified by cDNA transfection by ELISA.

**Supplementary Figure 5.**


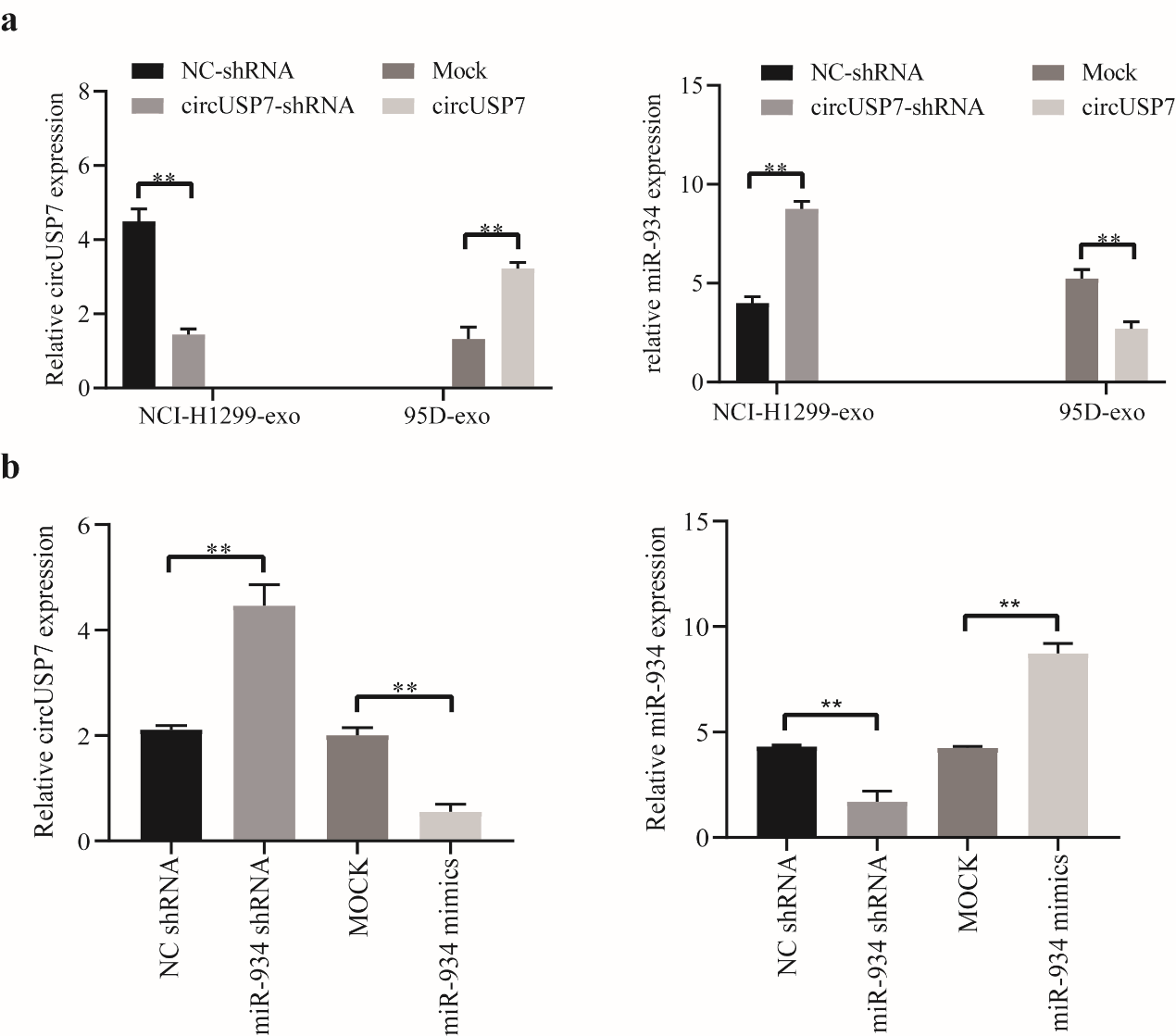


**Supplementary Figure 5. The relationship between circUSP7 and miR-934 expression in** **CD8+T cells**. **a** circUSP7 or miR-934 expression in CD8^+^T cells was co-cultured with exosome derived from NCI-H1299 cells was modified by transfection of shRNA and 95D cells were modified by cDNA transfection. **b** circUSP7 expression in CD8^+^T cells was modified by miR-934 mimics or shRNA transfection, miR-934 expression in miR-934-overexpressing or miR-934-silenced CD8^+^T cells. The data are presented as the mean ± SD. **P < 0.01.

**Supplementary Figure 6.**

**
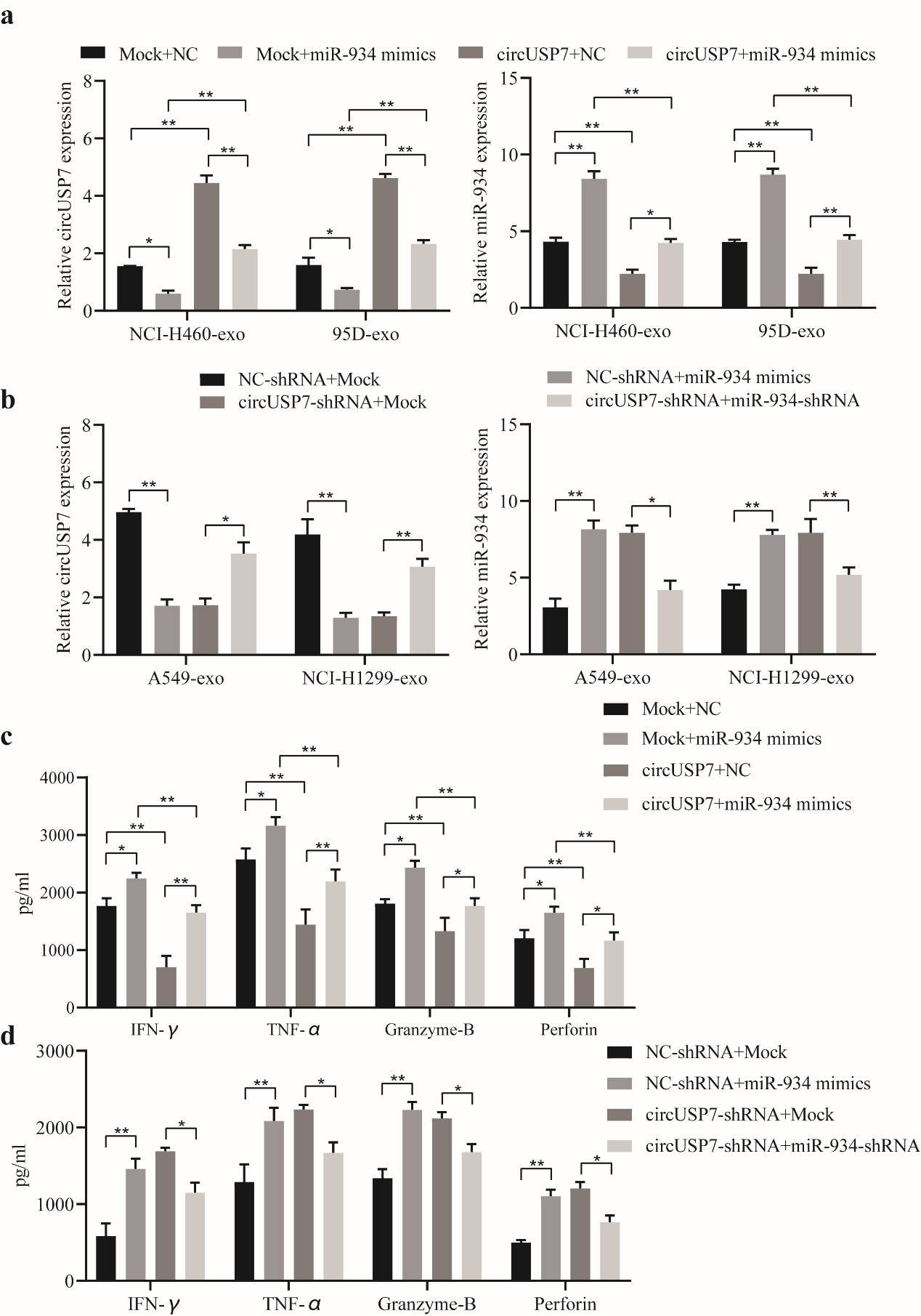
**

**Supplementary Figure 6. miR-934 affects the function of circUSP7 in CD8^+^T cells. a** The expression of circUSP7 and miR-934 in CD8^+^T cells co-cultured with exosomes derived from NCI-H460 and 95D cells was modified by transfection of shRNA, and modified with miR-934 expression. **b** The expression of circUSP7 and miR-934 in CD8^+^T cells co-cultured with exosomes derived from NCI-H1299 and A549 cells was modified by transfection of cDNA, and modified with miR-934 expression. **c** The secretion of IFN-γ, TNF-α, Perforin, and Granzyme-B expression in CD8+T cells co-cultured with exosomes derived from NCI-H460 cells was modified by cDNA transfection with modified circUSP7 and miR-934 expression. **d** The secretion of IFN-γ, TNF-α, Perforin, and Granzyme-B expression in CD8^+^T cells co-cultured with exosomes derived from A549 cells was modified by transfection of shRNA with modified circUSP7 and miR-934 expression.

**Supplementary Figure 7.**

**
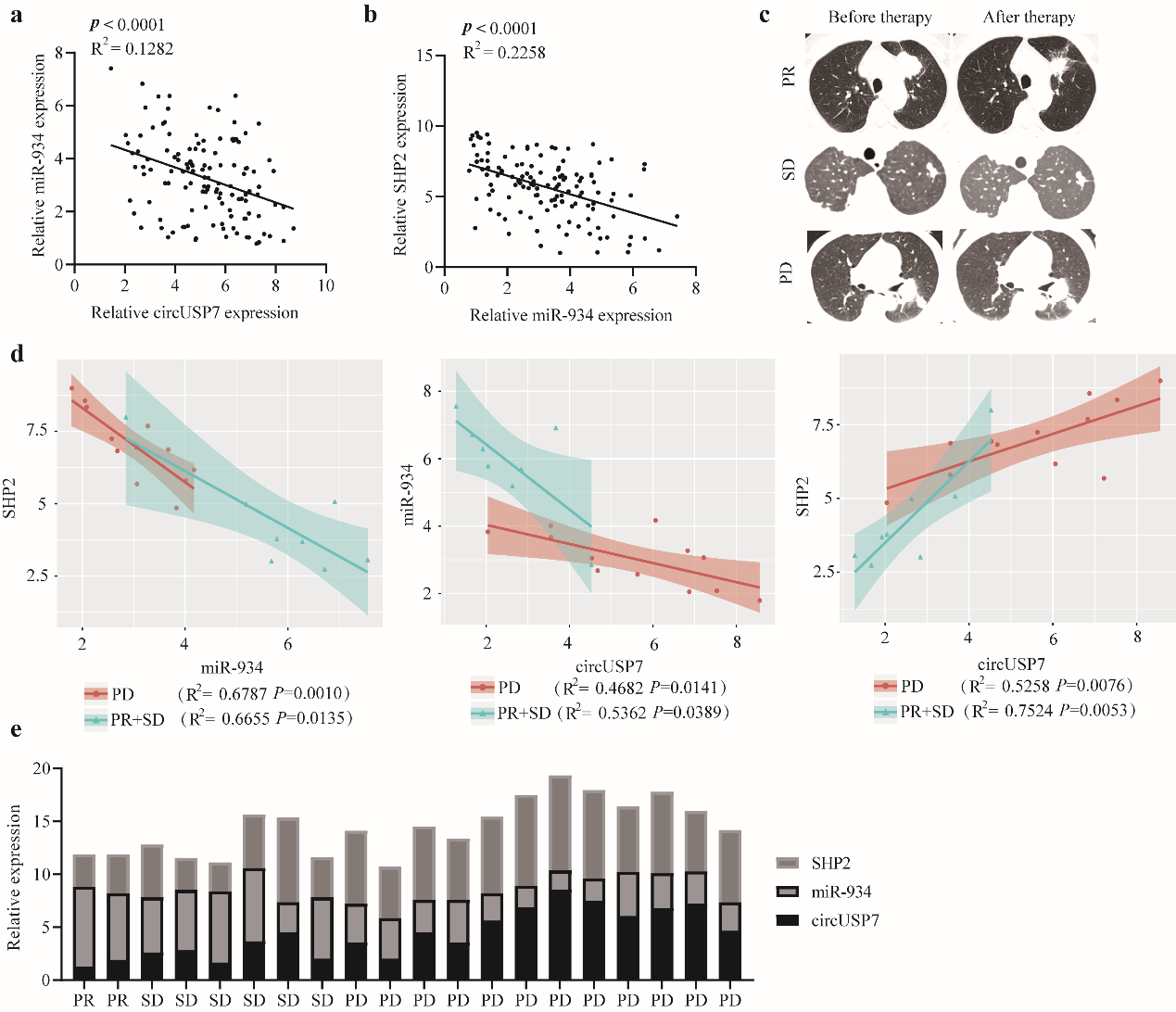
**

**Supplementary Figure 7. a** A negative correlation between circUSP7 and miR-934 was observed in the NSCLC tissues (R^2^ = 0.1282; P < 0.0001). **b** A negative correlation between SHP2 mRNA and miR-934 was observed in the NSCLC tissues (R^2^ = 0.2258; P < 0.0001). **c** The PD1 antibody immunotherapy efficacy assessment using CT-based RECIST1.1. **d** and **e** The relationship between circUSP7 and miR-934, SHP2 and circUSP7, and SHP2 and miR-934 expression in patients with PD or PR + SD after anti-PD1 therapy.

**Supplementary Figure 8.**


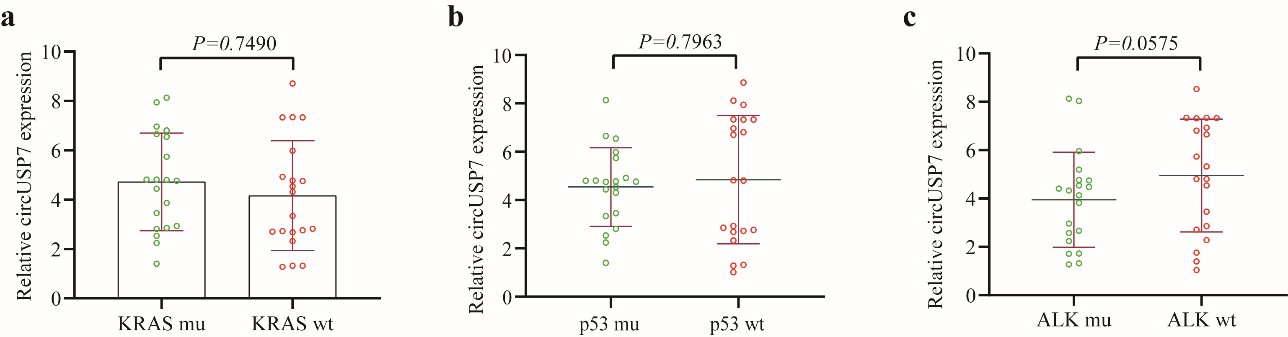


**Supplementary Figure 8.** the relationship between circUSP7 and Kras, p53 and ALK mutation status in NSCLC.
